# Letter from G. F. J. Colburn

**Published:** 1848-07

**Authors:** G. F. J. Colburn

**Affiliations:** Newark, N.J.


### ARTICLE X.

### *Letter from* G. F. J. Colburn, *Newark, N. J.*

Dr. Harris :—

Dear Sir :—Soon after the introduction of chloroform into this country, I discovered it would *readily dissolve gutta percha ;* forming a solution, which with the addition of a few drops of spirits turpentine, possessed considerable adhesiveness and became solid after a short exposure to the air, as well as being perfectly impervious to moisture and not injured by the atmosphere or acids. As this solution possessed such peculiar properties, the idea suggested itself of applying it for dental purposes—such as cementing plaster *casts* when broken, coating pivots and the surfaces of the root when inserting a pivot tooth, in order to shield the junction of the parts from the moisture and secretions of the mouth, &c.

I have used the preparation in numerous cases for the above purposes, also, for checking hemorrhage about the gums and mouth, and as a dressing for small incisions, and abrasions, &c. about the hands, and found it to answer an admirable purpose in all cases; for such uses I would recommend it to your notice. I send you some of the gutta percha, which you can dissolve by placing it in a bottle and adding chloroform to it—say four parts of the chloroform to one of spirits turpentine; the bottle should have a ground stopple to prevent the evaporation of the solvents.

I hope you will give this solution a trial, and if it should answer a good purpose, make it known to the profession through the medium of your journal.

